# Asymmetric response of vegetation NPP to extreme climate over a vulnerable karst Region in Southwest China

**DOI:** 10.1371/journal.pone.0360970

**Published:** 2026-10-27

**Authors:** Zhiyuan Yang, Jingjie Geng, Kang Xu, Fangfang Luo, Fengxia Tian

**Affiliations:** 1 School of Economics and Management, Tongren University, Tongren, China; 2 Engineering Center of Intelligent Monitoring and Policy Simulation of Mountainous Territory Space, Tongren, China; 3 Tongren Rural Revitalization Research Institute, Tongren, China; 4 Qiandong Agricultural (Village) Development and Ecological Governance Laboratory, Tongren, China; Central University of Rajasthan, INDIA

## Abstract

Understanding how vegetation in ecologically fragile karst regions responds to extreme climate events is critical for global change adaptation. However, the response of net primary productivity (NPP) to extreme climate indices, rather than to mean climatic conditions, has not been systematically investigated across large, contiguous karst areas. To address this gap, this study used MODIS NPP data and 12 ETCCDI extreme climate indices calculated from daily meteorological records (2000–2018) to investigate the spatiotemporal evolution of NPP and its asymmetric response to extreme temperature and precipitation events across Guizhou Province, Southwest China. Pixel-level Pearson correlation and Theil-Sen trend analyses were applied, with false discovery rate (FDR) correction for multiple comparisons. The results show that: (1) annual mean NPP increased significantly at a rate of approximately 3.8 g C m^−2^ yr^−1^, with a west-high, east-low spatial pattern; (2) climate trends showed asymmetric warming (nighttime warming three to four times faster than daytime) and intensified total precipitation but reduced persistence; and (3) NPP exhibited strong, spatially extensive positive correlations with extreme temperature indices (especially warm night frequency and annual minimum temperature), whereas correlations with extreme precipitation indices were weak and highly heterogeneous. Thermal conditions, particularly nighttime warming, appear to be the primary driver of vegetation dynamics in this humid karst region, while the unique dualistic hydrological structure likely decouples precipitation from plant water availability. Future karst ecosystem management should prioritize extreme temperature events and adopt spatially differentiated strategies.

## 1 Introduction

Increasingly frequent, intense, and prolonged extreme climate events characterize contemporary global change, posing severe threats to the structure and function of terrestrial ecosystems [1–3]. Vegetation net primary productivity (NPP), a pivotal measure of ecosystem carbon sequestration capacity, is central to understanding the global carbon cycle [3,4]. Consequently, elucidating how NPP responds to extreme climate has become a critical research frontier for projecting ecological impacts and designing effective adaptation strategies [5,6]. This need is particularly urgent in ecologically fragile regions, where such understanding is fundamental for assessing vulnerability and building climate resilience [7,8].

Climatic factors, especially hydrothermal conditions and soil moisture availability, are primary regulators of vegetation dynamics [9,10]. However, extreme climate events often impose more acute and direct impacts than changes in mean climatic conditions [8]. Compound extremes, such as concurrent heatwaves and droughts, are particularly damaging; their synergistic effects can stress ecosystems far beyond the impact of any single hazard [11]. Observations across China confirm a consistent rise in extreme climate indices, which exhibit distinct regional heterogeneity and present escalating risks to ecosystem stability [12,13]. In karst regions, these climate-vegetation interactions are further complicated by rapid soil water infiltration and anthropogenic disturbances (e.g., land conversion, overgrazing), creating feedback loops that remain poorly understood.

These climate-vegetation interactions are especially critical in karst ecosystems, which are renowned for their topographic complexity, unique hydrology, and high sensitivity to climate change [14]. The distinctive “dualistic three-dimensional” hydrological structure—epitomized by the phrase “soil upstairs, water downstairs”—allows rapid drainage of surface soil moisture through subsurface conduits, leaving vegetation highly susceptible to water stress despite seemingly sufficient rainfall [15,16]. While remote sensing studies generally report an increasing trend in NPP across southwestern China’s karst region [17–19], and some have linked these dynamics to conventional climate factors like temperature and evapotranspiration [15], a critical knowledge gap remains. A systematic understanding of how NPP responds specifically to extreme climate indices—rather than conventional climatic means—is still lacking. This gap is particularly concerning given the unique hydrological characteristics of karst that may decouple precipitation from plant water availability.

Most existing studies rely on regional averages or a limited set of extreme indices, thereby failing to capture the full spatiotemporal heterogeneity of the response or to quantitatively map the spatial coupling between NPP and a comprehensive suite of extreme climate indicators [20,21]. For instance, conclusions from non-karst basins [22] cannot be directly extrapolated due to fundamentally different hydrological and ecological processes. Moreover, research findings have rarely been translated into spatially explicit guidance for management, limiting their practical application in combating rocky desertification or informing programs like Grain-for-Green [23,24].

To address these gaps, this study focuses on Guizhou Province, the core area of the world’s largest contiguous karst region in southwestern China. Guizhou provides an ideal natural laboratory for three main reasons. First, its extensive karst landscape (61.9% of the provincial area) forms a distinctive dualistic hydrological system that strongly decouples surface water availability from precipitation. Second, the province lies within a subtropical humid monsoon climate zone and is frequently affected by extreme climatic events. Third, long-term MODIS NPP datasets and high-density meteorological station observations provide a robust basis for spatially explicit analysis.

Based on the above, this study tests three hypotheses: (i) nighttime warming (as indicated by TN90p and TMIN) has a stronger positive effect on NPP than daytime warming (TX90p and TMAX); (ii) due to the dualistic hydrological structure of karst, NPP does not show a significant or spatially coherent response to extreme precipitation indices; and (iii) there exists a clear west-east spatial differentiation in NPP trends, corresponding to differences in ecological restoration intensity and inherent karst vulnerability. Using remote sensing and meteorological data from 2000 to 2018, this study aims to: (1) characterize the spatiotemporal dynamics of vegetation NPP and 12 and extreme climate indices; (2) quantify the pixel-level correlation and asymmetric response of NPP to extreme temperature and precipitation indices; and (3) explore the underlying mechanisms and discuss their implications for karst ecosystem management under climate change.

## 2 Materials and methods

### 2.1 Study area

Guizhou Province (24°37′–29°13′ N, 103°36′–109°35′ E) constitutes a critical ecological barrier in the upper reaches of the Yangtze and Pearl Rivers in southwestern China (Fig 1). The terrain, characterized by plateaus, mountains, and hills, slopes from west to east. The region experiences a typical subtropical humid monsoon climate, with a mean annual temperature of approximately 15°C and annual precipitation ranging from 1000 to 1400 mm. A defining feature is its extensive karst landscape, covering 61.9% of the province and making it one of the world’s largest contiguous karst areas [25]. The distinctive karst geology fosters a unique dualistic hydrological structure (“soil upstairs, water downstairs”), which heightens ecosystem sensitivity to hydrological variations. The region’s susceptibility to compound extreme climate events, including seasonal droughts and short-duration heavy rainfall [26–29], makes it an ideal natural laboratory for investigating vegetation responses to climate extremes.

**Fig 1:**
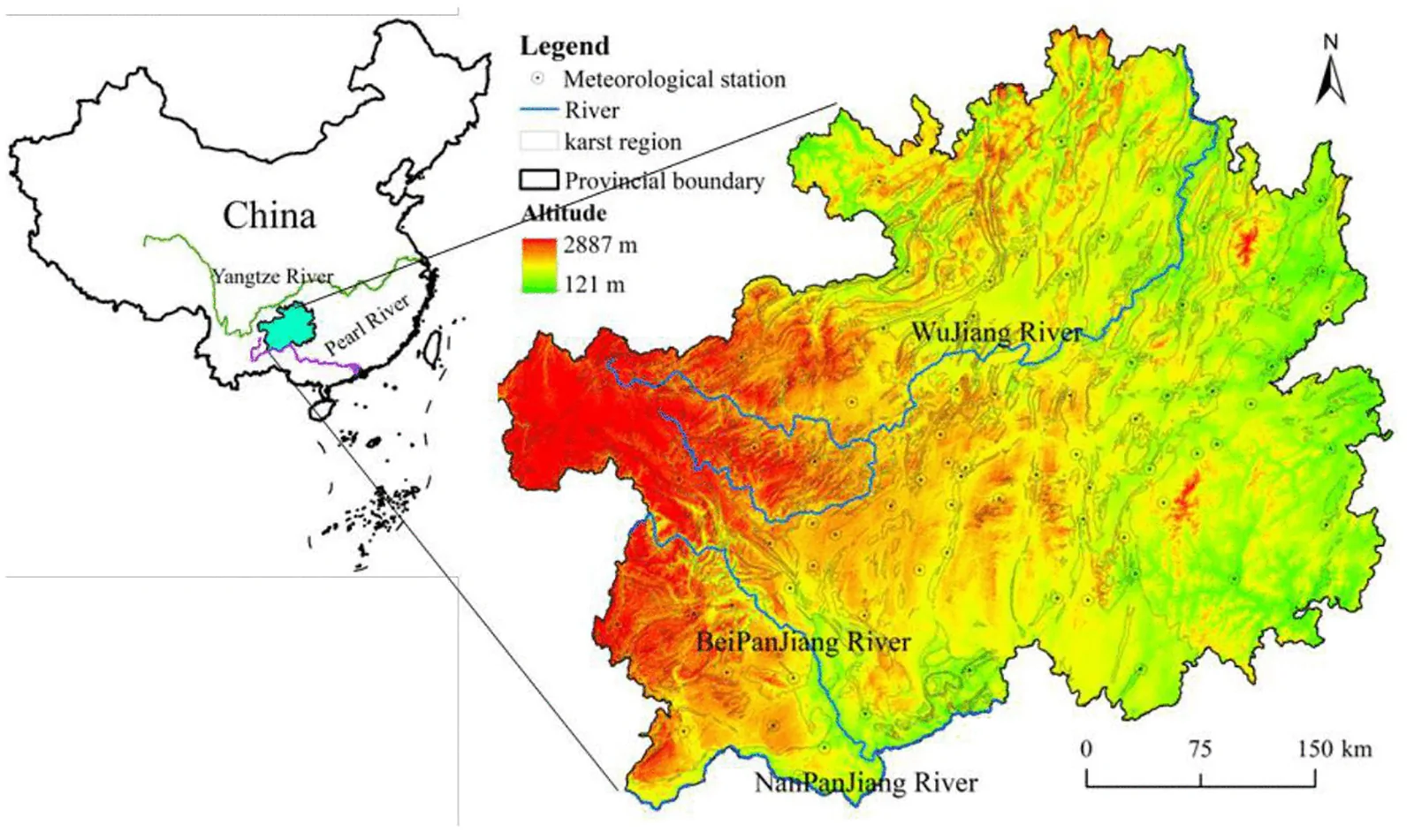
Location of the study area. The base map was obtained from Natural Earth Data (public domain; https://www.naturalearthdata.com/). The study area boundary, elevation data, and station locations were compiled specifically for this study using SRTM DEM data (NASA JPL, https://doi.org/10.5067/MEaSUREs/SRTM/SRTMGL1.003) and administrative boundary data from the Resource and Environment Science and Data Center (https://www.resdc.cn/).

### 2.2 Data sources and processing

#### 2.2.1 Meteorological data and extreme climate indices.

Daily meteorological data (maximum temperature, minimum temperature, precipitation) for the period 2000–2018 were obtained from the “Daily Value Dataset of Surface Climate Data in China (V3.0)” provided by the China Meteorological Data Service Center (https://data.cma.cn/) [30]. From an initial pool of 84 national standard stations in and around Guizhou Province, 55 stations with complete and high-quality records over the study period were selected for analysis.

Rigorous quality control was implemented, involving the identification and removal of outliers (values exceeding 3 standard deviations from the climatological mean). For the minimal missing data (typically 1–2 days at individual stations), interpolation was performed using the average of values from adjacent days. For instances of consecutive missing days(up to 5 days), gaps were filled using the station’s climatological mean for the corresponding calendar dates, calculated from the 2000–2018 base period. Following the guidelines of the Expert Team on Climate Change Detection and Indices (ETCCDI), 27 core extreme climate indices were computed using the RClimDex package (version 1.1) in R (R Core Team, 2020), capturing the intensity, frequency, and duration of extreme events. Finally, the annual values of these indices at each station were spatially interpolated into continuous 500-m resolution raster datasets using ordinary Kriging(exponential variogram model), ensuring compatibility with the NPP data for pixel-level analysis.

The selection of 12 indices (6 temperature, 6 precipitation) from the full set of 27 ETCCDI indices was based on (i) their frequent use in vegetation-climate studies in South China, (ii) minimal multicollinearity (variance inflation factor <5 after stepwise elimination), and (iii) relevance to vegetation physiological processes (e.g., warm nights affect respiration, heavy precipitation affects soil erosion). The final set of 12 indices showed acceptable collinearity (mean VIF = 2.3, maximum VIF = 4.1).

#### 2.2.2 Vegetation Net Primary Productivity (NPP) Data.

The annual vegetation NPP data for 2000–2018 were sourced from the MOD17A3HGF Version 6.1 product(https://doi.org/10.5067/TERRA/MOD17A3HGF.061), distributed by NASA’s EarthData platform. This product, derived from a light use efficiency model, provides estimates of vegetation carbon sequestration capacity at an annual temporal resolution and 500-meter spatial resolution. It is acknowledged that MOD17A3HGF has inherent uncertainties in heterogeneous karst terrain (e.g., mixed pixels, steep slopes), but it remains the most consistent dataset for long-term regional trend analysis.

The raw data files were first mosaicked and converted to the desired format. The projection was then unified to the Albers Equal Area Conic projection (based on the WGS84 datum) to maintain area accuracy for spatial calculations. Finally, the data were clipped to the boundary of Guizhou Province to generate the final time-series NPP dataset for this study.

### 2.3 Methodology

#### 2.3.1 Extreme climate index calculation.

From the full set of 27 ETCCDI indices, 12 key indices (6 for temperature, 6 for precipitation) were selected for detailed analysis. Their definitions are provided in Table 1. All indices were calculated using the standardized RClimDex 1.1 software [31].

**Table 1:** Definitions of the extreme climate indices used in this stud.

Category	Index Name	Unit	Description
Extreme Precipitation	Consecutive Dry Days (CDD)	days	Max number of consecutive days with RR < 1 mm
	Consecutive Wet Days (CWD)	days	Max number of consecutive days with RR ≥ 1 mm
	Annual total precipitation (PRCPTOT)	mm	Sum of annual precipitation from wet days (RR ≥ 1 mm)
	Number of heavy precipitation days (R10mm)	days	Annual count of days when RR ≥ 10 mm
	Very wet days precipitation (R95p)	mm	Annual total precipitation when RR > 95th percentile
	Max 1-day precipitation (RX1day)	mm	Annual maximum 1-day precipitation
Extreme Temperature	Annual mean max temp (TMAX)	°C	Mean of daily maximum temperatures
	Annual mean min temp (TMIN)	°C	Mean of daily minimum temperatures
	Cold night frequency (TN10p)	%	Percentage of days when TN < 10th percentile
	Warm night frequency (TN90p)	%	Percentage of days when TN > 90th percentile
	Cold day frequency (TX10p)	%	Percentage of days when TX < 10th percentile
	Warm day frequency (TX90p)	%	Percentage of days when TX > 90th percentile

Note: RR = daily precipitation; TN = daily minimum temperature; TX = daily maximum temperature. Percentiles are calculated based on the 2000–2018 base period.

#### 2.3.2 Trend analysis.

The temporal trends of regionally averaged NPP and extreme climate indices were assessed using univariate linear regression. The trend slope a in the linear model y = ax + b (where y is the variable and x is the year) represents the mean annual change rate [32].

To analyze the spatial trend of NPP, the non-parametric Theil-Sen median estimator was employed due to its robustness against outliers. The trend slope β at each pixel is calculated as the median of all pairwise slopes between different years:


$$\begin{array}{c} \begin{array}{c} \beta =\text{Median}\left(\frac{\left({\text{x}}_{\text{j}}-{\text{x}}_{\text{i}}\right)}{\left(\text{j}-\text{i}\right)}\right),\;\forall \text{j}>\text{i} \end{array} \end{array}$$ (1)

where xi and xj are the NPP values in years i and j, respectively. A positive β indicates an increasing trend [33].

#### 2.3.3 Correlation analysis.

The pixel-based linear relationship between interannual variations in NPP and each extreme climate index was quantified using the Pearson correlation coefficient (r). The coefficient for each pixel was calculated as:


$$\begin{array}{c} \begin{array}{c} {\text{r}}_{\text{xy}}=\frac{\sum_{\text{i}=\text{1}}^{\text{n}}[({\text{x}}_{\text{i}}-\overset{―}{\text{x}})({\text{y}}_{\text{i}}-\overset{―}{\text{y}})]}{\sqrt{\sum_{\text{i}=\text{1}}^{\text{n}}{\left({\text{x}}_{\text{i}}-\overset{―}{\text{x}}\right)}^{\text{2}}\sum_{\text{i}=\text{1}}^{\text{n}}{\left({\text{y}}_{\text{i}}-\overset{―}{\text{y}}\right)}^{\text{2}}}} \end{array} \end{array}$$ (2)

where xi and xj represent the NPP value and the extreme climate index value in year i, respectively; $\overset{―}{\text{x}}$ and $\overset{―}{\text{y}}$ are their respective means over the study period; and n is the number of years. The value of r ranges from −1–1, To account for multiple comparisons, the Benjamini–Hochberg false discovery rate (FDR) procedure was applied at a family- wise error rate of q < 0.05. Only correlations that passed the FDR threshold are reported as statistically significant.

#### 2.3.4 Statistical significance testing.

The statistical significance of the observed trends(for regionally averaged time series) was assessed using the non-parametric Mann-Kendall (M-K) test [34,35], which is robust against non-normally distributed data and outliers. The test statistic S is computed as:


$$\begin{array}{c} \begin{array}{c} \text{S}=\sum_{\text{i}=\text{1}}^{\text{n}-\text{1}}\sum_{\text{j}=\text{i}+\text{1}}^{\text{n}}\text{sgn}\left({\text{x}}_{\text{j}}-{\text{x}}_{\text{i}}\right) \end{array} \end{array}$$ (3)

where xi and xj are the sequential data values, n is the length of the time series, and the sign function sgn(z) is defined as:


$$\begin{array}{c} \begin{array}{c} \;\text{sgn}(\text{Z})=\left\{ \begin{array}{c} @r\begin{array}{cc} \text{1}, & {\text{ x}}_{\text{j}}-{\text{x}}_{\text{i}}>\text{0} \end{array}\\ \begin{array}{cc} \text{0}, & \;{\text{x}}_{\text{j}}-{\text{x}}_{\text{i}}=\text{0} \end{array}\\ \begin{array}{cc} -\text{1}, & {\text{x}}_{\text{j}}-{\text{x}}_{\text{i}}<\text{0}\; \end{array} \end{array}\right. \end{array} \end{array}$$ (4)

For series with n ≥ 10, the statistic S is approximately normally distributed. Its variance is given by:


$$\begin{array}{c} \begin{array}{c} \text{Va}\text{r}\left(\text{S}\right)=\frac{\text{n}\left(\text{n}-\text{1}\mathrm{\backslash rightleft}(\text{2n}+\text{5}\right)}{\text{18}} \end{array} \end{array}$$ (5)

The standardized test statistic Z is then derived as follows for hypothesis testing:


$$\begin{array}{c} \begin{array}{c} \text{Z}=\left\{ \begin{array}{c} @r\begin{array}{cc} \frac{\text{s}-\text{1}}{\sqrt{\text{Var}\left(\text{S}\right)}}, & \text{if s}>\text{0} \end{array}\\ \begin{array}{cc} \text{0}, & \text{if s}=\text{0} \end{array}\\ \begin{array}{cc} \frac{\text{s}+\text{1}}{\sqrt{\text{Var}\left(\text{S}\right)}}, & \text{if s}<\text{0} \end{array} \end{array}\right. \end{array} \end{array}$$ (6)

A positive (negative) Z value indicates an increasing (decreasing) trend. The trend was considered statistically significant at the 95% confidence level if |Z| > 1.96 [36]. For pixel–level trends, the same M–K test was applied but with FDR correction for multiple testing.

Additionally, the significance of the Pearson correlation coefficients was evaluated using a two-tailed t-test. Correlations were deemed significant at p < 0.05 after applying the FDR correction.

## 3 Results

### 3.1 Spatiotemporal evolution of vegetation NPP

From 2000–2018, NPP in Guizhou Province exhibited a significant upward trend, with annual value ranging from approximately 705–843 g C m^−2^ yr^−1^ (Fig 2a). The mean annual NPP was 782 g C m^−2^ yr^−1^, with the maximum in 2013 and the minimum in 2000. A notable decline occurred between 2010 and 2012, which may be associated with consecutive extreme cold events.

**Fig 2:**
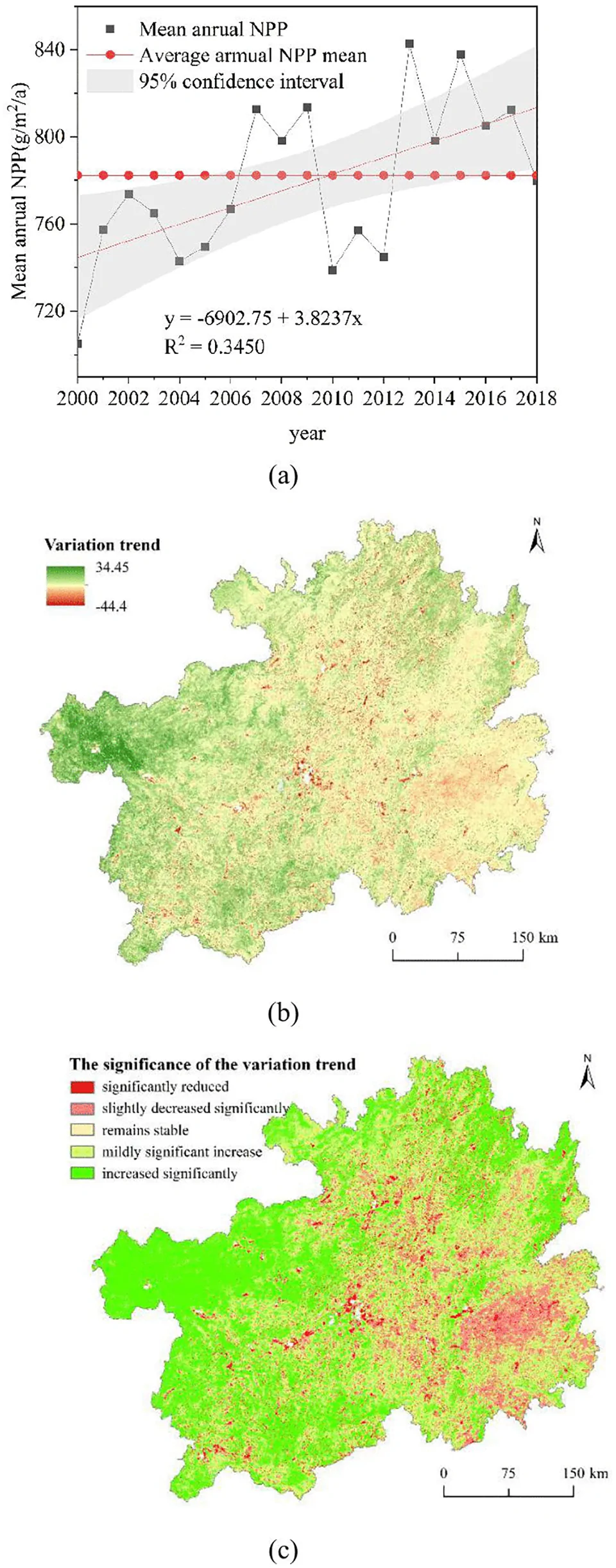
Spatiotemporal patterns of vegetation NPP in Guizhou Province from 2000 to 2018. (a) Interannual variation of the provincial mean NPP (g C m^−2^ yr^−1^), with the linear trend line (red) and 95% confidence interval (shaded). (b) Spatial distribution of the multi-year mean NPP (g C m^−2^ yr^−1^). (c) Spatial pattern of NPP trends estimated by Theil–Sen median estimator (g C m^−2^ yr^−1^). Colors represent different trend categories (red: significantly reduced; pink: slightly decreased; yellow: remains stable; green: mildly significant increase; light green: increased significantly). Base maps for the spatial distributions are from Natural Earth Data (public domain).

Spatially, the largest NPP gains occurred in western regions, while central and eastern areas showed stable or slightly declining trends (Fig 2b and 2c). Statistically, 80.56% of the province’s area showed increasing NPP trends; 48.85% of those increases were significant (p < 0.05 after FDR correction), predominantly in the west and southwest (Fig 2c). Areas of decreasing NPP covered 19.34% of the province, with 20.27% of this decrease being significant, primarily in the central region.

### 3.2 Spatiotemporal evolution of extreme climate indices

#### 3.2.1 Extreme precipitation indices.

From 2000 to 2018, extreme precipitation indices showed an increase in total annual precipitation (PRCPTOT) and heavy precipitation (R95p), reflecting enhanced intensity. However, the persistence of precipitation events, measured by consecutive dry days (CDD) and consecutive wet days (CWD), showed a decreasing trend. Temporally, significant increases were observed for PRCPTOT (+30.15 mm/decade, p < 0.05) and R95p (+26.72 mm/decade, p < 0.05) (Fig 3). In contrast, CDD (–0.62 days/decade) and CWD (–0.48 days/decade) showed non‑significant decreases. RX1day displayed a slight, non‑significant increasing trend (+2.80 mm/decade).

**Fig 3:**
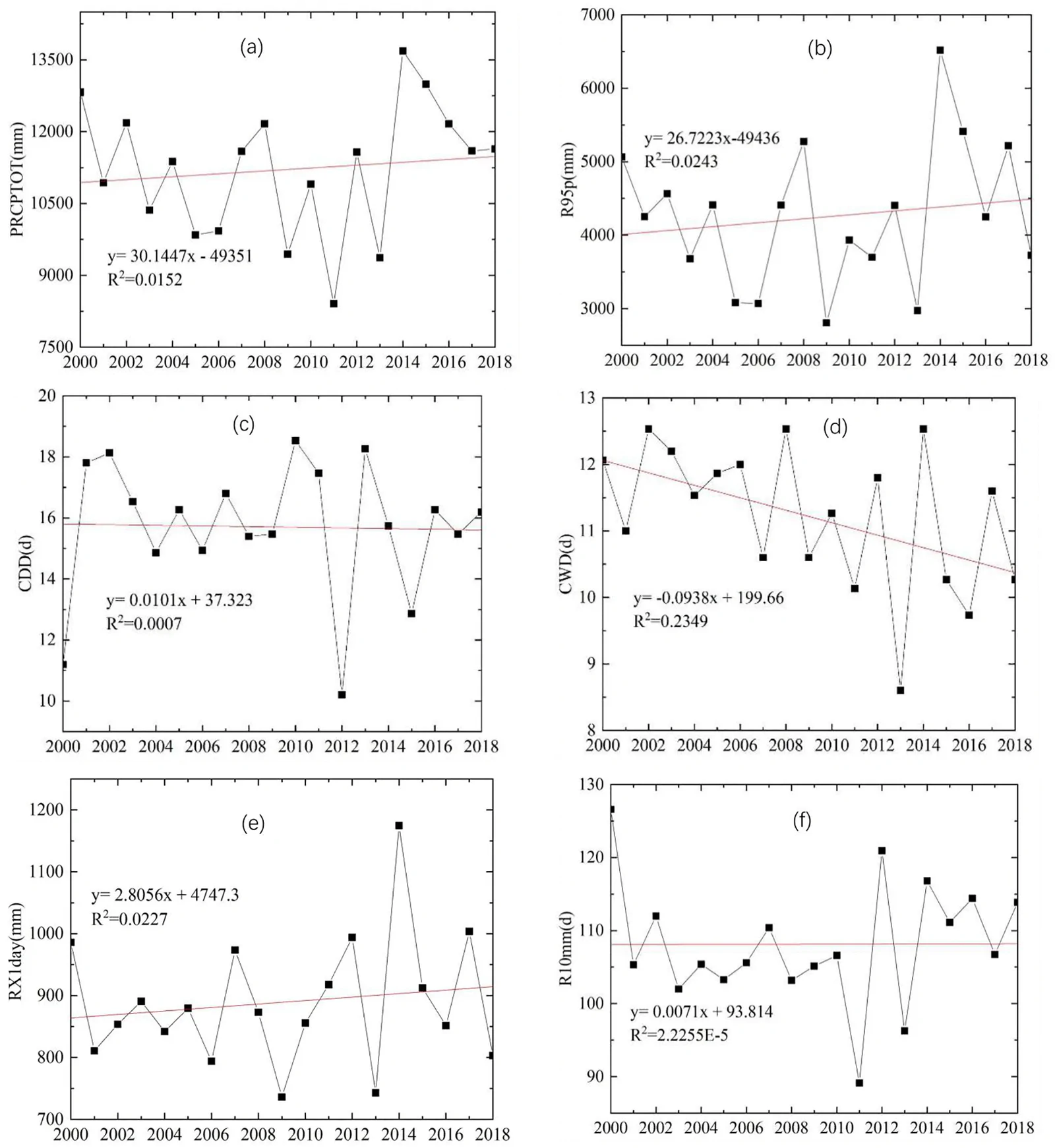
Temporal trends of extreme precipitation indices (2000–2018). Each panel shows the annual time series (blue dots) and linear trend line (red) for: (a) PRCPTOT (mm), (b) R95p (mm), (c) RX1day (mm), (d) R10mm (days), (e) CDD (days), (f) CWD (days). Slope values and p‑values (from M–K test) are indicated in each panel.

Spatially, the evolution of extreme precipitation indices exhibited clear heterogeneity (Fig 4). Significant increasing trends for PRCPTOT and R95p were mainly concentrated in the southeastern part of Guizhou Province. The increasing trend for RX1day was most evident in the eastern regions. CDD showed a non-significant increasing trend in the central and eastern parts, while areas of significant decrease for CWD were concentrated in the west and southwest. This spatial pattern of —increased precipitation in the southeast and decreased precipitation persistence in the southwest—may be related to the combined effects of monsoon circulation changes and complex topography.

**Fig 4:**
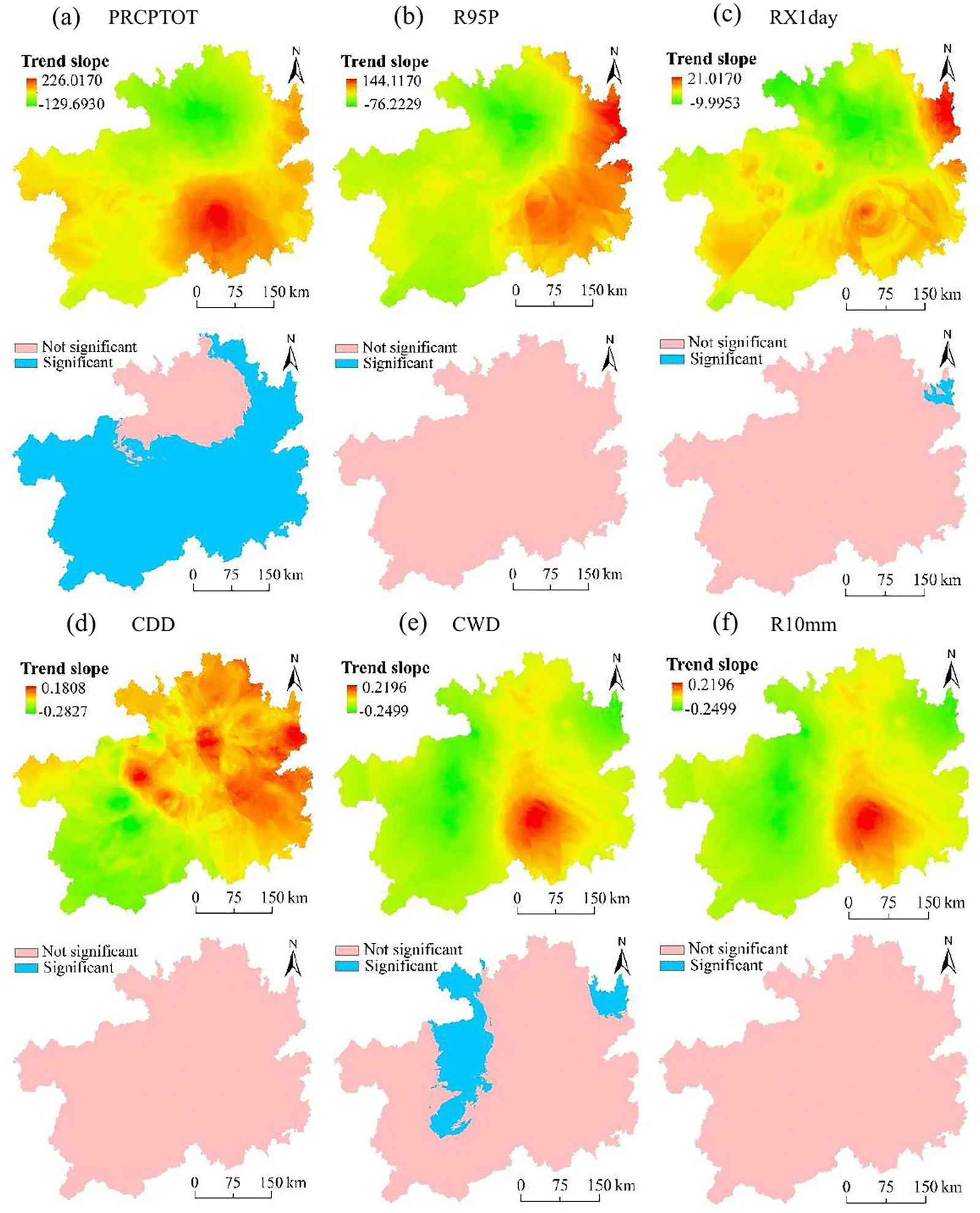
Spatial Patterns of Trends and Statistical Significance of extreme precipitation indices. Each map corresponds to an index: (a) PRCPTOT, (b) R95p, (c) RX1day, (d) CDD, (e) CWD, (f) R10mm. Colors indicate Theil‑Sen slope magnitude (mm/decade for PRCPTOT, R95p, RX1day; days/decade for others). Hatched areas indicate significant trends (p < 0.05 after FDR correction). Base map from Natural Earth Data (public domain).

#### 3.2.2 Extreme temperature indices.

During the study period, extreme temperature indices consistently pointed towards a significant warming trend, exhibiting asymmetric warming characteristics: the rate of nighttime warming was higher than daytime warming, and the rate of decrease in cold extremes was greater than the rate of increase in warm extremes.

In the time series (Fig 5), the warm night frequency (TN90p) increased sharply at a rate of 3.6 days/decade, much faster than the warm day frequency (TX90p, 0.9 days/decade, p = 0.08). Cold night frequency (TN10p) decreased at a notably faster rate (−3.1 days/decade, p < 0.01) than cold day frequency (TX10p, −0.8 days/decade, p = 0.12). The mean annual minimum temperature (TMIN) rose at 0.42 °C/decade (p < 0.01), approximately four times that of mean annual maximum temperature (TMAX, 0.11 °C/decade, p = 0.21).

**Fig 5:**
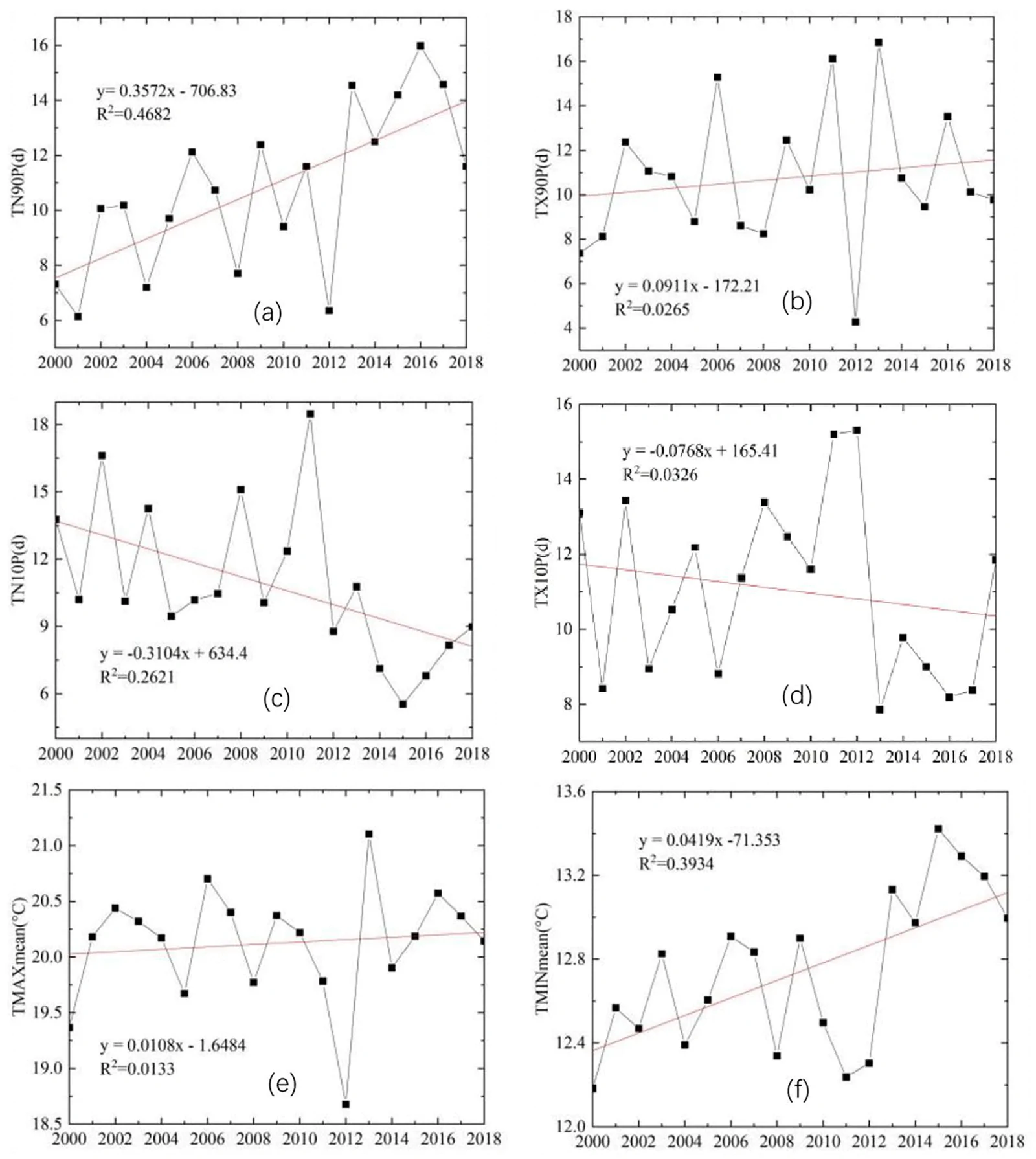
Temporal trends of extreme temperature indices (2000–2018). Each panel shows the annual time series (red dots for warm indices, blue dots for cold indices) and linear trend line (black) for: (a) TMAX (°C), (b) TMIN (°C), (c) TX90p (days), (d) TN90p (days), (e) TX10p (days), (f) TN10p (days). Slope values and p‑values (from M–K test) are indicated in each panel.

Spatially, the warming trend was widespread but varied in intensity (Fig 6). Areas of significant increase for TN90p and TMIN were widely distributed, particularly prominent in the northern, northeastern, and western regions. In contrast, significant increase areas for TX90p and TMAX were relatively smaller, mainly concentrated in the central and northeastern parts. Areas of significant decrease for TX10p and TN10p were primarily distributed in the western and southwestern regions. This spatial pattern may be related to the interactive effects of altitude, urbanization processes, and aerosol emissions.

**Fig 6:**
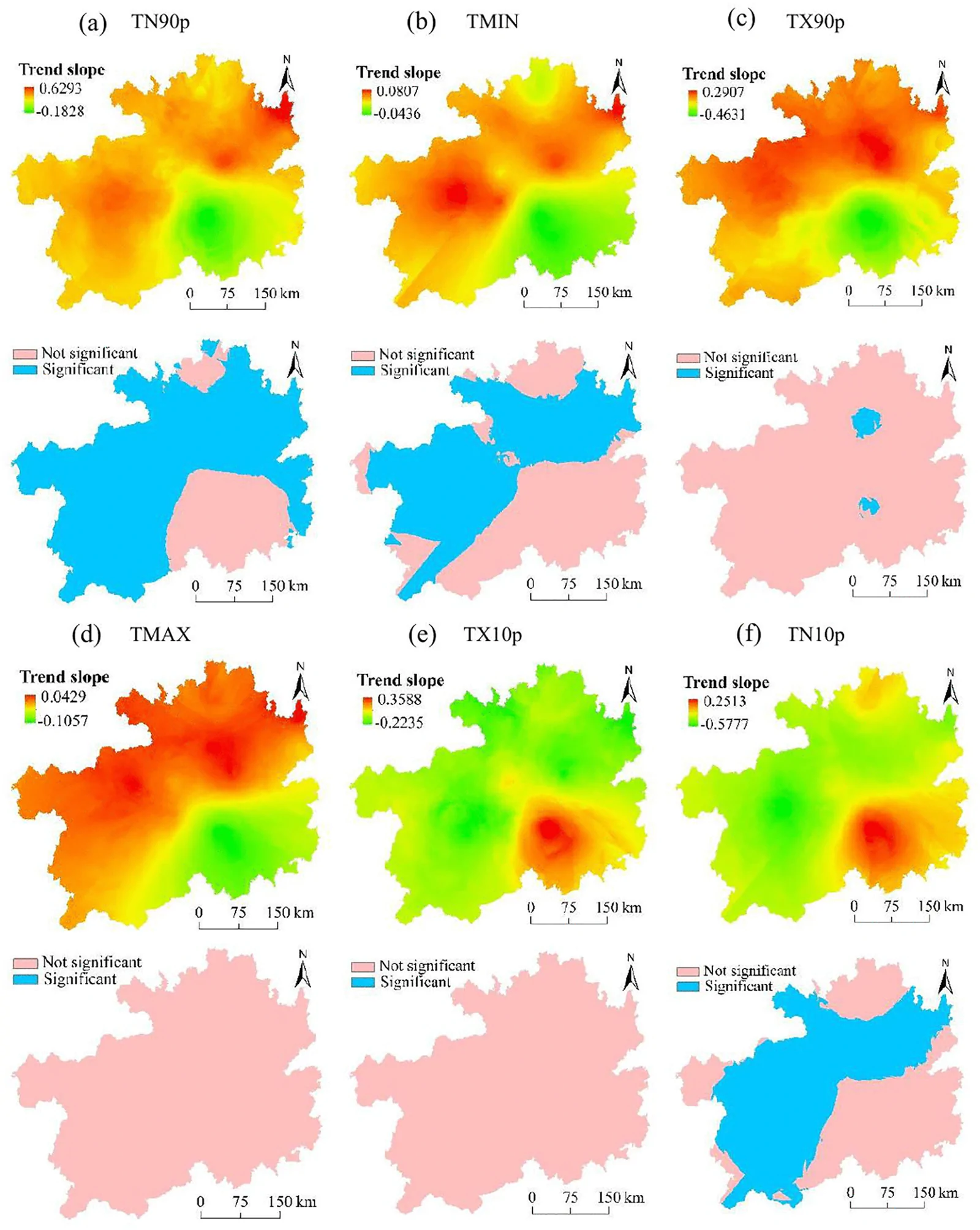
Spatial Patterns of Trends and Statistical Significance of extreme temperature indices. Each map corresponds to an index: (a) TN90p, (b) TMIN, (c) TX90p, (d) TMAX, (e) TX10p, (f) TN10p. Colors indicate Theil‑Sen slope magnitude (°C/decade for TMAX, TMIN; days/decade for others). Hatched areas indicate significant trends (p < 0.05 after FDR correction). Base map from Natural Earth Data (public domain).

### 3.3 Response of vegetation NPP to extreme climate indices

#### 3.3.1 Responses of NPP to extreme precipitation indices.

Correlations between NPP and most extreme precipitation indices were generally weak and spatially heterogeneous, with no discernible dominant pattern (Fig 7). Positive correlation areas (based on the sign of r, before significance filtering) accounted for 26.8% of pixels for R10mm and 51.9% for R95p; these were interwoven with negative correlation areas, and only a sparse distribution of pixels passed the 95% significance test. These results indicate that the impact of moderate-to-heavy precipitation on vegetation growth is not universally deterministic but is likely mitigated or enhanced by local contingencies such as topography and soil moisture redistribution.

**Fig 7:**
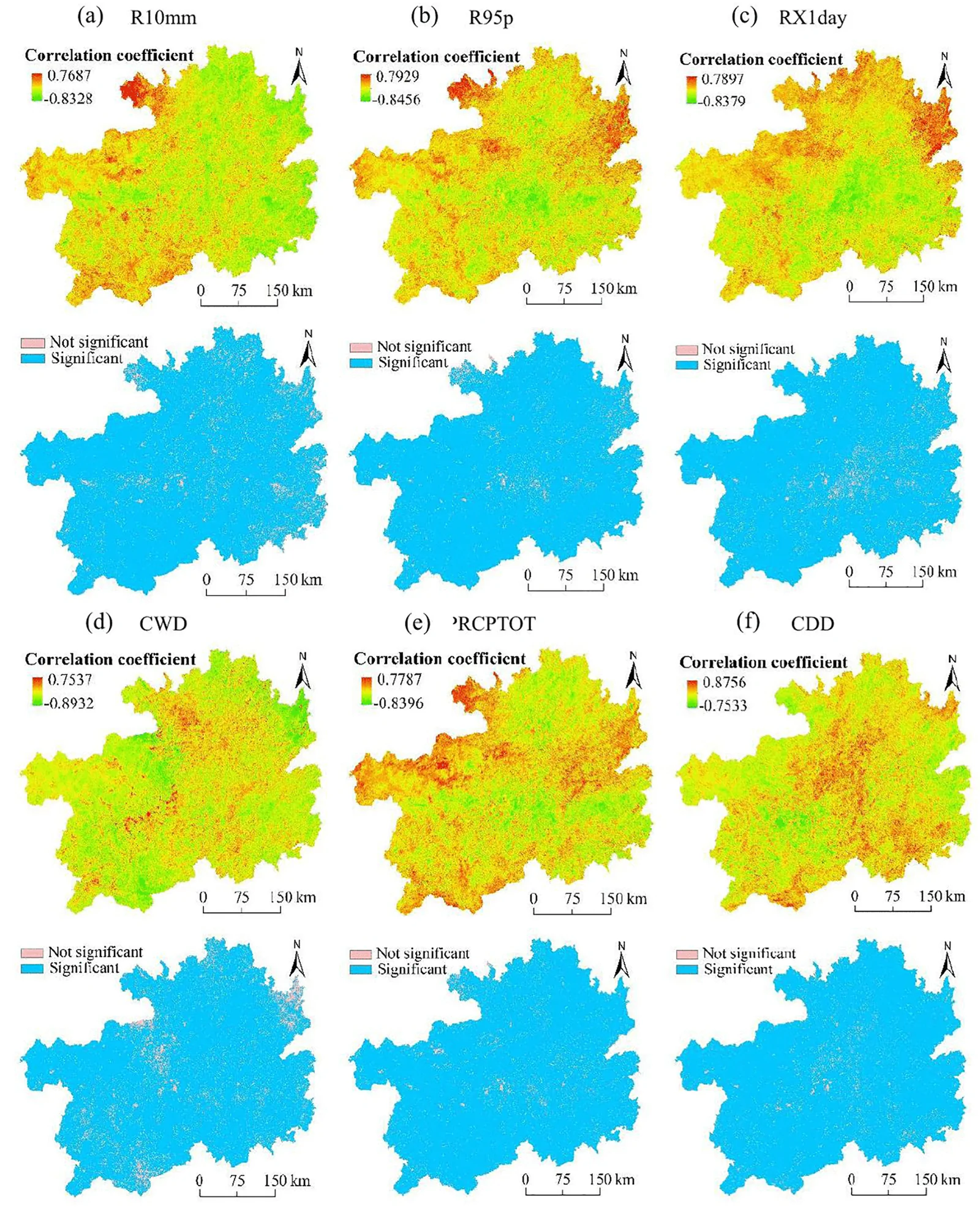
Spatial Patterns of Pearson Correlation Coefficients and statistical significance between NPP and extreme precipitation indices. Each map corresponds to an index: (a) R10mm, (b) R95p, (c) RX1day, (d) CWD, (e) PRCPTOT, (f) CDD. Colors indicate correlation coefficient r. Hatched areas indicate significant correlations after FDR correction (q < 0.05). Base map from Natural Earth Data (public domain).

A negative correlation between RX1day and NPP was observed in 61.9% of the region, with 26.8% of these pixels significant after FDR correction. For CWD, 84.0% of the area showed negative correlation, with 50.2% significant. In contrast, PRCPTOT showed a weak positive correlation in 53.9% of the region, with only 17.3% significant. This divergence highlights the potential compensatory roles of total rainfall amount versus prolonged wet spells, with the latter likely limiting NPP by attenuating photosynthetically active radiation.

#### 3.3.2 Response to extreme temperature events.

In contrast, NPP exhibited strong, spatially extensive, and statistically significant positive correlations with extreme temperature indices (Fig 8). Positive correlations between NPP and TMAX occurred in 85.6% of pixels, and with TMIN in 85.8% of pixels, with substantial contiguous areas passing the FDR‑corrected significance test. For relative indices, NPP was predominantly positively correlated with warm indices (TX90p: 78.9%; TN90p: 86.8%) and negatively correlated with cold indices (TX10p: 86.5%; TN10p: 76.1%). The positive correlation for warm nights (TN90p) was the most spatially extensive and statistically significant.

**Fig 8:**
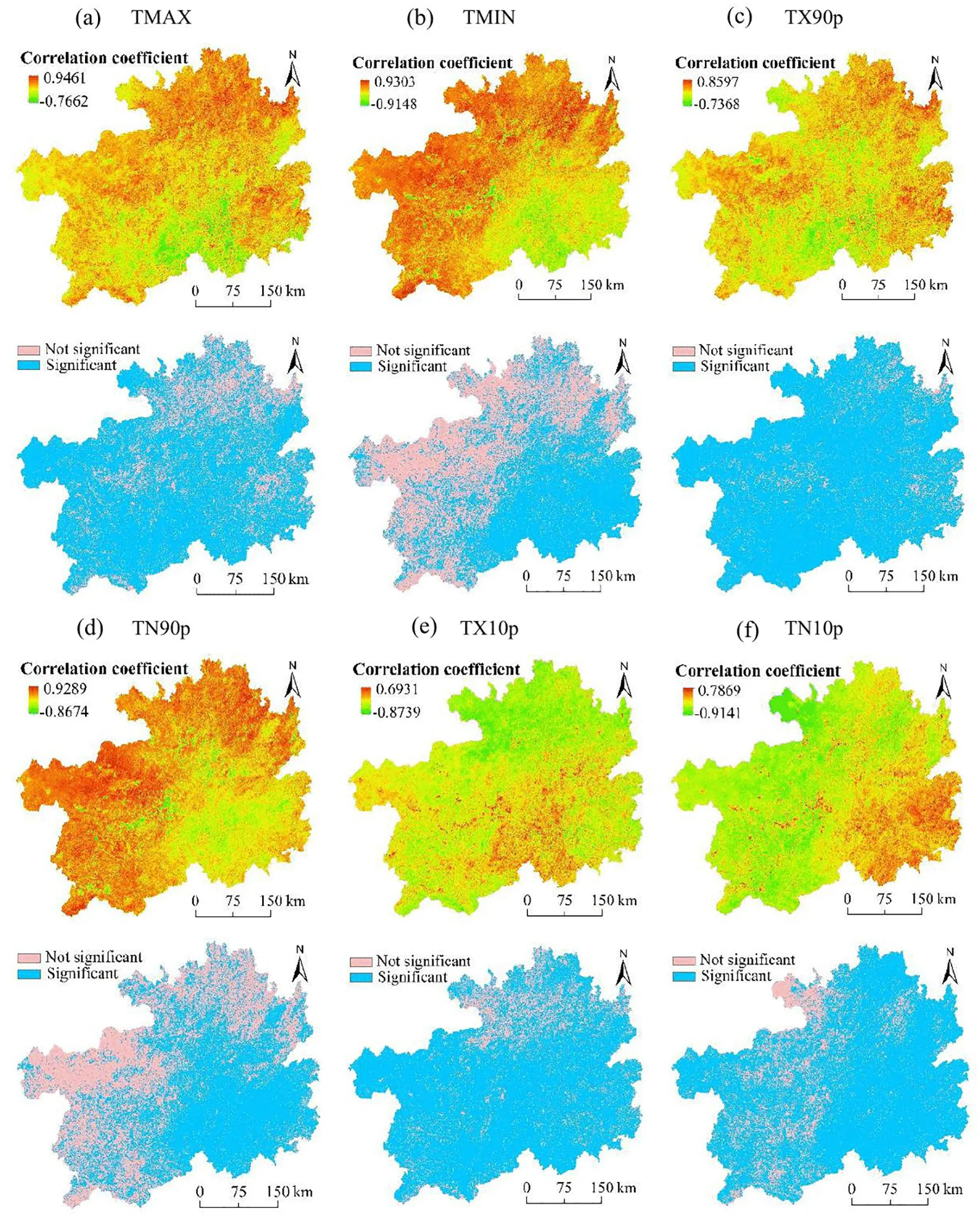
Spatial Patterns of Pearson Correlation Coefficients and statistical significance between NPP and extreme temperature indices. Each map corresponds to an index: (a) TMAX, (b) TMIN, (c) TX90p, (d) TN90p, (e) TX10p, (f) TN10p. Colors indicate correlation coefficient r Hatched areas indicate significant correlations after FDR correction (q < 0.05). Base map from Natural Earth Data (public domain).

## 4 Discussion

### 4.1 Drivers of NPP increase and spatial heterogeneity

Vegetation NPP in Guizhou Province increased significantly from 2000 to 2018, aligning with broader regional greening trends [37,38]. The strong positive correlations with TMIN and TN90p suggest that climate warming is a major driver. In this subtropical karst ecosystem, rising temperatures likely alleviate cold stress and extend the growing season, indicating that thermal—rather than moisture—limitations dominate [39,40]. Spatially, the largest NPP gains occurred in areas where ecological restoration projects (e.g., Rocky Desertification Control) have been implemented, which may indicate a positive anthropogenic influence [41,42]; however, this study did not quantitatively separate the effects of climate versus human activities. In contrast, growth stagnation in central and eastern regions highlights the modulating role of local factors, such as inherent karst vulnerability and human pressure. These findings underscore that regional gains can mask local degradation risks [43].

### 4.2 Mechanisms behind the asymmetric response

A key finding is the asymmetric NPP response: strongly positive to extreme temperatures but weak and heterogeneous to extreme precipitation.

#### 4.2.1 Dominance of thermal limitation.

The robust positive correlation with extreme temperature indices suggests that heat is a primary climatic driver of NPP in this region [44]. Nocturnal warming (TN90p, TMIN) appears particularly influential, likely reducing nighttime respiration and frost damage, thereby promoting net growth [44,45]. This effect is more pronounced than daytime warming (TX90p, TMAX), which is mechanistically plausible: warmer nights decrease respiratory carbon loss, alleviate low‑temperature constraints on dark reactions, and reduce frost damage to meristems—all directly enhancing net carbon accumulation. Previous studies have also reported asymmetric effects of daytime vs. nighttime warming on vegetation [44].

#### 4.2.2 Modulation by karst hydrology.

The weak NPP response to precipitation reflects karst hydrological characteristics. Rapid infiltration in these landscapes limits surface water availability despite increased rainfall [14–16]. Short-duration heavy rainfall (RX1day) provides minimal benefit and may even induce soil erosion, while consecutive wet days (CWD) can inhibit growth by reducing photosynthetically active radiation (PAR) [46]. Consequently, karst hydrology effectively “filters” precipitation signals, preventing a coherent regional response. This interpretation is consistent with previous studies that reported low sensitivity of NPP to climatic factors in karst regions [19].

### 4.3 Comparison with previous studies

These findings refine earlier work in Southwest China. Zhang et al. [47] emphasized NPP’s sensitivity to temperature, but this study highlights nocturnal warming as a critical factor. The negative NPP-CWD relationship supports hypotheses that excess rainfall may inhibit vegetation growth in karst areas [48]. Divergences with studies emphasizing precipitation [49] may reflect differences in spatial scales and the focus on extreme events rather than mean conditions. In non‑karst basins, such as loess regions, surface soil moisture retention enhances NPP–precipitation coupling, whereas in karst regions, rapid infiltration decouples them. This contrast underscores the fundamental influence of karst hydrology on climate‑vegetation interactions. Similar decoupling effects have been reported in other karst regions globally (e.g., Mediterranean karst, Southeast Asian karst), suggesting that the hydrological filtering mechanism may be a general feature of karst ecosystems.

### 4.4 Limitations and future research

This study has several limitations. First, while MODIS NPP data are widely used, ground‑based validation would strengthen confidence, especially in heterogeneous karst terrain. Second, spatial interpolation of climate data (ordinary Kriging) may smooth local extremes. Third, correlation does not imply causation; the observed associations suggest but do not prove causal relationships. Process‑based modeling (e.g., LPJ‑GUESS, Biome‑BGC) is needed to establish causal mechanisms. Fourth, human activities (e.g., afforestation, urbanization, agricultural management) were not explicitly separated from climatic effects. Fifth, the 19‑year period (2000–2018) may not represent long‑term trends beyond this interval. Future research should integrate high‑resolution soil moisture and groundwater data, along with detailed human activity information, to disentangle climate–anthropogenic interactions more effectively.

### 4.5 Implications for management

These findings provide actionable insights for adaptive ecosystem management: Western NPP hotspots: Maintain conservation status and prevent fragmentation. Central/eastern stagnation zones: Prioritize rocky desertification control and soil retention measures. Forestry adaptation: Introduce heat‑ and drought‑tolerant native species and adjust planting schedules to coincide with later warm nights. Water management: Despite weak NPP–precipitation correlations, small‑scale water harvesting structures can exploit intense rainfall events and mitigate seasonal droughts exacerbated by karst hydrology.

Given that these management recommendations are based on the 2000–2018 climate–vegetation relationships, their applicability under future climate change should be periodically reassessed.

## 5 Conclusions

This study systematically reveals the spatiotemporal dynamics of vegetation net primary productivity (NPP) and its response to extreme climate in Guizhou Province from 2000 to 2018.

Annual NPP increased significantly at a rate of 3.82 g C m^−2^ yr^−1^, exhibiting a distinct spatial pattern of higher NPP in the west and lower NPP in the east. The western region acted as a hotspot for carbon sequestration, while growth stagnation in the central and eastern regions reflected the combined effects of topography and human activities.

Extreme climate trends were characterized by predominant warming and altered precipitation regimes. A pronounced asymmetric warming was observed, marked by intensified nocturnal warming (TN90p, TMIN) and a decline in extreme cold events. Meanwhile, precipitation trends indicated a shift toward more concentrated and intense events.

A pronounced asymmetry was identified in the climatic drivers: NPP exhibited strong, widespread positive correlations with extreme temperature indices, unequivocally confirming thermal conditions as the primary climatic controller. In stark contrast, the response to extreme precipitation was weak and spatially heterogeneous, consistent with the interpretation that the unique karst hydrology decouples precipitation from plant water availability.

These findings underscore the dominance of asymmetric warming in promoting vegetation growth in this humid karst region. They necessitate climate adaptation strategies that prioritize thermal factors and implement spatially differentiated conservation to sustain regional carbon sinks.
